# New diagnosis in psychiatry: beyond heuristics

**DOI:** 10.1017/S003329172400223X

**Published:** 2025-02-06

**Authors:** Patrick D. McGorry, Ian B. Hickie, Roman Kotov, Lianne Schmaal, Stephen J. Wood, Sophie M. Allan, Kürşat Altınbaş, Niall Boyce, Laura F. Bringmann, Avshalom Caspi, Bruce Cuthbert, Łukasz Gawęda, Robin N. Groen, Sinan Guloksuz, Jessica A. Hartmann, Robert F. Krueger, Cristina Mei, Dorien Nieman, Dost Öngür, Andrea Raballo, Marten Scheffer, Marieke J. Schreuder, Jai L. Shah, Johanna T. W. Wigman, Hok Pan Yuen, Barnaby Nelson

**Affiliations:** 1 Orygen, Parkville, VIC, Australia; 2 Centre for Youth Mental Health, The University of Melbourne, Parkville, VIC, Australia; 3 Brain and Mind Centre, University of Sydney, Sydney, NSW, Australia; 4 Department of Psychiatry, Stony Brook University, Stony Brook, New York, USA; 5 School of Psychology, University of Birmingham, Birmingham, UK; 6 Norwich Medical School, University of East Anglia, Norwich, UK; 7 Department of Psychiatry, Selcuk University Faculty of Medicine, Konya, Turkey; 8 Wellcome, London, UK; 9 Department of Psychometrics and Statistics, University of Groningen, Groningen, The Netherlands; 10 Interdisciplinary Center of Psychopathology and Emotion Regulation (ICPE), University Medical Center Groningen, University of Groningen, Groningen, The Netherlands; 11 Department of Psychology and Neuroscience, Duke University, Durham, NC, USA; 12 Department of Psychiatry and Behavioral Sciences, Duke University School of Medicine, Durham, NC, USA; 13 Center for Genomic and Computational Biology, Duke University, Durham, NC, USA; 14 Social, Genetic, and Developmental Psychiatry Research Centre, Institute of Psychiatry, Psychology, and Neuroscience, King’s College London, London, UK; 15 PROMENTA Center, University of Oslo, Oslo, Norway; 16 National Institute of Mental Health, Bethesda, MD, USA; 17 Experimental Psychopathology Lab, Institute of Psychology, Polish Academy of Sciences, Warsaw, Poland; 18 Department of Psychiatry and Neuropsychology, School for Mental Health and Neuroscience, Maastricht University Medical Center, Maastricht, The Netherlands; 19 Department of Psychiatry, Yale School of Medicine, New Haven, CT, USA; 20 Department of Public Mental Health, Medical Faculty Mannheim, Central Institute of Mental Health, Heidelberg University, Heidelberg, Germany; 21 Department of Psychology, University of Minnesota, Minneapolis, MN, USA; 22 Department of Psychiatry, Amsterdam University Medical Centers, Amsterdam, The Netherlands; 23 Department of Psychiatry, McLean Hospital, Harvard Medical School, Belmont, MA, USA; 24 Chair of Psychiatry, Faculty of Biomedical Sciences, Università della Svizzera Italiana, Lugano, Switzerland; 25 Cantonal Socio-psychiatric Organization, Public Health Division, Department of Health and Social Care, Repubblica e Cantone Ticino, Mendrisio, Switzerland; 26 Wageningen University, Wageningen, The Netherlands; 27 Department of Psychiatry, McGill University, Montreal, QC, Canada; 28 Prevention and Early Intervention Program for Psychosis (PEPP), Douglas Mental Health University Institute, Montreal, QC, Canada; 29 ACCESS Open Minds, Douglas Mental Health University Institute, Montreal, QC, Canada

**Keywords:** Psychiatric Diagnosis, Paradigm Shift, Research Domain Criteria (RDoC), the Hierarchical Taxonomy of Psychopathology (HiTOP), Clinical Staging, Network Analysis, Complex Systems, Clinical Utility

## Abstract

**Background:**

Diagnosis in psychiatry faces familiar challenges. Validity and utility remain elusive, and confusion regarding the fluid and arbitrary border between mental health and illness is increasing. The mainstream strategy has been conservative and iterative, retaining current nosology until something better emerges. However, this has led to stagnation. New conceptual frameworks are urgently required to catalyze a genuine paradigm shift.

**Methods:**

We outline candidate strategies that could pave the way for such a paradigm shift. These include the Research Domain Criteria (RDoC), the Hierarchical Taxonomy of Psychopathology (HiTOP), and Clinical Staging, which all promote a blend of dimensional and categorical approaches.

**Results:**

These alternative still heuristic transdiagnostic models provide varying levels of clinical and research utility. RDoC was intended to provide a framework to reorient research beyond the constraints of DSM. HiTOP began as a nosology derived from statistical methods and is now pursuing clinical utility. Clinical Staging aims to both expand the scope and refine the utility of diagnosis by the inclusion of the dimension of timing. None is yet fit for purpose. Yet they are relatively complementary, and it may be possible for them to operate as an ecosystem. Time will tell whether they have the capacity singly or jointly to deliver a paradigm shift.

**Conclusions:**

Several heuristic models have been developed that separately or synergistically build infrastructure to enable new transdiagnostic research to define the structure, development, and mechanisms of mental disorders, to guide treatment and better meet the needs of patients, policymakers, and society.

## Critique: diagnosis in psychiatry

Diagnosis in medicine is viewed as an essential process in choosing appropriate treatment, predicting illness course, and providing clarity and relief to patients that their illness is legitimate and understood. The relationship between disease, causation, and diagnosis is complex, and the diagnostic process operates on several levels (Scadding, 1996). In many fields of medicine, the diagnostic process has evolved in complexity over the past few decades from a predominantly clinical and phenotypic process to *precision medicine* (Collins & Varmus, 2015), which places a great deal of weight on investigations aimed at staging and stratifying or personalizing treatment in a more precise manner. This evolution is focused on improving the utility of diagnosis. Mainstream psychiatric research has embraced this paradigm; however, it remains almost completely aspirational.

A review of the historiography of psychiatric diagnosis and classification reveals several alternative theoretical approaches to defining the underlying nature of psychiatric disorders. None have led to a model that is fit for purpose. The current approach based on DSM and ICD superficially resembles the approach of standard medical diagnosis, yet we are no closer to a precision medicine, in which specific mechanisms and therapeutic targets play a meaningful role. This aspiration constantly seems tantalizingly within reach but has so far proven to be a mirage. One obstacle, often minimized, derives from the reality of heterogeneity and pleiotropism (McGorry, 1991a, b). Syndromes in medicine, as final common pathways, are underpinned by a range of underlying pathophysiological mechanisms. Pleiotropism reflects the converse, namely that any single pathophysiological process gives rise to a range of syndromes. These often evolve through a series of stages. The substantial disconnect that remains between current diagnostic frameworks and validity and clinical utility continues to dilute their value.

At the same time, the effects of medical diagnosis are powerful, deceptively complex, and there are significant risks as well as potential benefits (Lea & Hofmann, 2022). In psychiatry, the benefits have not only been more elusive, but the risks more pronounced. Furthermore, the balance between risks and benefits varies across the diagnostic spectrum. Some diagnoses tend to be rejected because of their harmful effects, while others, notably Autism and ADHD, are being embraced with a degree of contagion, in pursuit of perceived benefits within a changing socioeconomic context. In general, attitudes to diagnosis in psychiatry remain ambivalent and polarized, reflecting Cartesian tensions between the extremes of ‘mindless’ and ‘brainless’ psychiatry (Angell, 2011). These tensions are reflected in the range of historical perspectives on the nature of psychiatric disorders that have been thoroughly rehearsed over the past century. Stein and colleagues and Kendler have recently provided erudite expositions of these perspectives (Kendler, 2016; Stein et al., 2024).

While this philosophical discourse continues, disillusionment has grown from a lack of utility of the existing diagnostic framework for treatment selection, and the overpromise and under-delivery of an excessively reductionist biological psychiatry. Current diagnostic models also represent an insufficient and relatively weak basis for allocating health care funding, and other indicators of complexity and treatment needs have become more salient (IHACPA, 2023). What is needed to transcend this impasse? Should we aim to build slowly and incrementally on the status quo (Stein et al., 2022), or aim for a paradigm shift? If any paradigm shift is to succeed, it must be built on sustainable scientific foundations. A global plan and change management process would need to be conducted in relation to the real-world impacts and challenges of replacing the highly embedded DSM and ICD frameworks with a different paradigm should one emerge. Such a paradigm would need to be comprehensively road-tested prior to stepwise and widespread adoption.

### Diagnosis as passport

Medicine in general and psychiatry in particular remain boundary managers: border police examining and certifying transit documents in an unceasing battle over depression and anxiety, sexuality and addiction. Psychiatry remains the peculiar legatee of such problems, an obligate participant in every generation’s particular cultural negotiations—a kind of canary at the pitface of cultural strife. (Rosenberg, 2006)

The border between mental health and mental illness is a soft border. It is readily crossed often without being aware of a transition and is difficult to map and define. The border has been shrouded in stigma, is under continual pressure from cultural, financial, and legal influences (Rosenberg, 2006), and is guarded by arbitrary diagnostic criteria and unyielding triage systems. The latter combine to restrict and exclude access to the neglected, underfunded, and overwhelmed systems of mental health care. The current reality is a hard border, with harmful effects, such as the exclusion of many who would benefit from treatment, and delays in treatment at earlier stages, which increases the risks of coercive forms of care and reduces the chances of recovery. A soft border may also pose dangers, notably stigma and the risk of premature and overdiagnosis. However, most of these potential harms can be overcome through healthy cultures of care and staged and proportional treatment. Overdiagnosis due to softening boundaries has surged in some domains, notably ADHD, ASD, and common mental disorders (Kazda et al., 2021; Mojtabai, 2013; Rødgaard et al., 2019). The harmful impact here is that such trends divert resources from those with genuine need and there may be a need for ‘dediagnosis’ in some areas (Lea & Hofmann, 2022; The Economist, 2023).

It is possible that well-intentioned awareness programs have softened this boundary and fuelled an extension of diagnosis beyond the point where it benefits people’s health (Foulkes, 2022; Lea & Hofmann, 2022). However, a soft and flexible border has many advantages, notably enabling early intervention and the patient to have a say in when help is sought. At least half, or perhaps the great majority, of us will experience at least one period of mental ill-health (Caspi et al., 2020; McGrath et al., 2023). It might be optimal to negotiate milder episodes of ill-health with a low-intensity or even a wait-and-see approach drawing upon self-help, social and peer support, or online help where available, at least for a short period. But there is no more reason than with physical ill-health, such as chest pain or a respiratory infection, to discourage or delay help-seeking at a primary care level. Early diagnosis, safe and proportional or staged intervention, depends on tolerance for such a soft border. Diagnosis can still be withheld or deferred, and people can be ‘dediagnosed’ around such a border too. In a positive sense, a reimagined diagnostic system should function as the patient’s passport for this border crossing.

### Diagnosis as useful

Diagnosis is essentially *classification with utility* (Kendell & Jablensky, 2003). The aim is to characterize the clinical phenotype in a shorthand way that helps to distinguish those who are ill and in need of care from those who are not, and enhance treatment selection and prognosis. A soft border creates some space for this as well as guarding against potential overdiagnosis, notably diagnoses that fail to provide any benefit and may cause harm (Lea & Hofmann, 2022).

**Figure 1.**
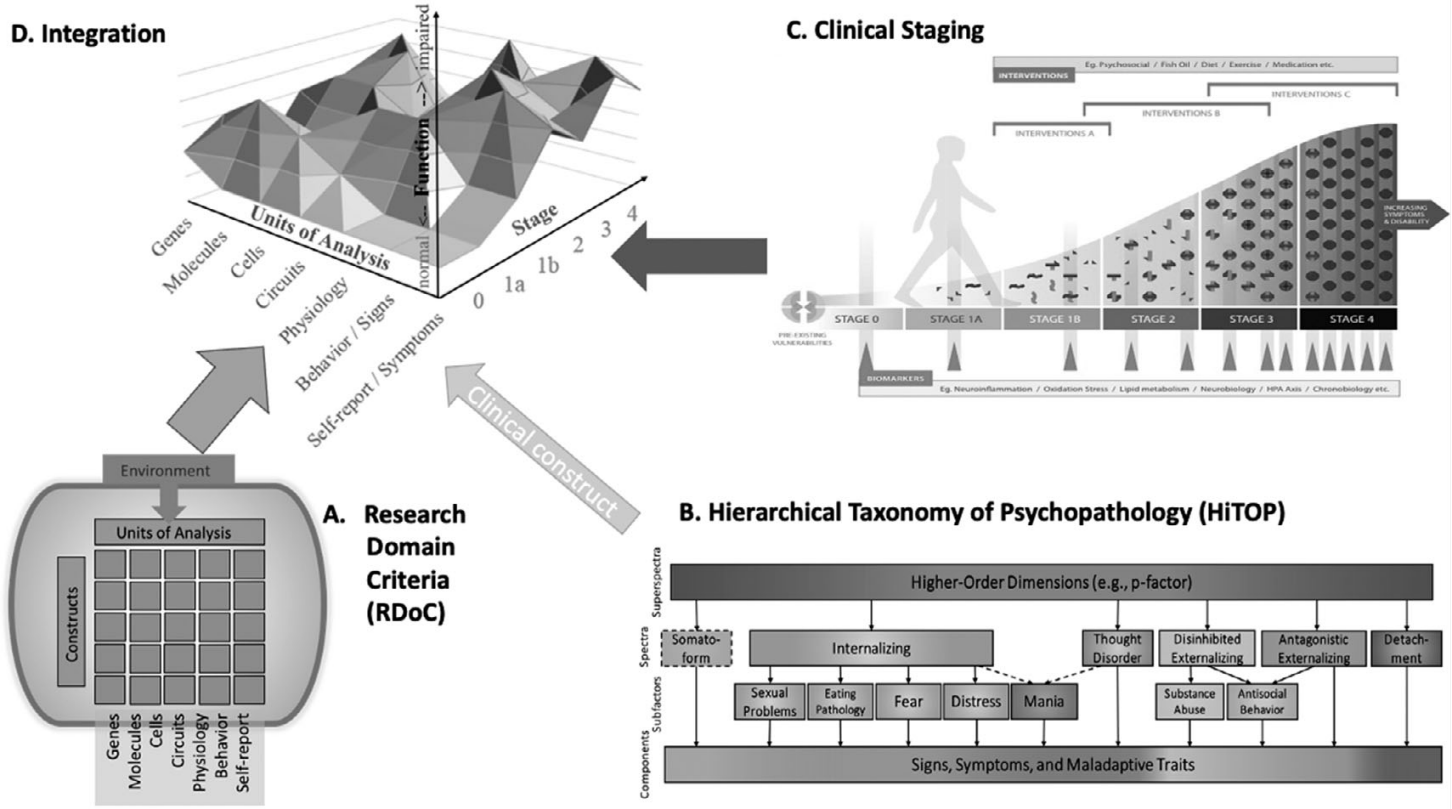
Alternative models and their integration. Panel A depicts the RDoC matrix of constructs (concepts representing a specified functional dimension of behavior) and seven units of analysis. Panel B depicts the HiTOP hierarchical organization of symptoms and maladaptive behaviors into progressively more general dimensions. Panel C illustrates the clinical staging model with the potential trajectory from asymptomatic state to late-stage severe and persistent mental illness with possible links to biomarkers. Panel D illustrates a conceptual integration of these models. The figure integrates time with evolution of the clinical phenotype by stage and different elements of neurobiology. A subset of individuals will progress from one stage to the next and some may remit.

Broad diagnostic categories are usually of limited utility. Therefore, some form of subclassification, to the extent that this sharpens treatment selection and prediction of outcome, has become essential to greater utility. *Staging* is one example of subclassification, where illness progression is defined according to subsequent stages of illness (Figure 1) (McGorry & Hickie, 2019; McGorry et al., 2006). Another example, compatible with and an enhancement of staging, is *stratification* through the definition of neurobiological and psychological subtypes that offer differing drug and treatment targets (Trusheim et al., 2007). This increasing precision means that the treatment options may be personalized in a relatively fine-grained manner (Collins & Varmus, 2015). However, there has always been a tension in psychiatric diagnosis between ‘lumping’ and ‘splitting’ and the basis for this has been somewhat arbitrary. The hierarchies of HiTOP (Hierarchical Taxonomy of Psychopathology; Figure 1), with the unitary ‘p’ factor at the apex, lump and split according to patterns of coherence within and between a finite number of dimensions of psychopathology. However, this form of lumping and splitting is mathematically based, according to the degree of statistical coherence and stability of symptom clusters, which may or may not map on to treatment response or prognosis. Ultimately, defining subcategories through precision or personalized medicine and therefore therapeutic utility would be the most useful form of splitting. This type of profiling based in part on biomarkers is highly compatible with, and can potentially redefine, a staging framework. It should also evolve with advances in research and treatment.

### Spurious precision

The advent of operational definition of putative disorders from the 1970s led to a marked improvement in reliability, at least in research settings. However, the definition of disorders and their boundaries was constrained by history and conservative opinion. DSM-IV produced a substantial expansion in the number of disorders constructed by committees, which blended opinions into consensus, albeit based on relatively sparse new evidence (Frances, 2013; Wakefield, 2016). Validity and utility continued to be conspicuously lacking. The spurious precision created has led to disillusionment and diminishing value of such nosologies in clinical practice (McGorry, 2010).

If we restrict ourselves to a purely psychopathological and syndromal level of analysis, the boundaries can be drawn widely or narrowly, and the ebbs and flows, the lumping and splitting, of the past 130 years will continue. Other challenges with this approach relate to the fact that the boundaries are obscure. There is a lack of ‘points of rarity’ between syndromes, nearly all operational definitions of syndromes are polythetic, and comorbidity is ubiquitous and managed inconsistently (McGrath et al., 2020). Furthermore, currently known biosignatures map poorly on to current diagnostic categories (Abi-Dargham et al., 2023), and almost certainly will require a transdiagnostic approach to map and align (Caspi & Moffitt, 2018; McGorry & van Os, 2013). To move beyond this impasse, different conceptual frameworks and new knowledge are required.

### Incrementalism vs paradigm shift

The crisis consists precisely in the fact that the old is dying and the new cannot be born. (Antonio Gramsci)

Stein et al. (2022) recently addressed the issue of diagnosis in an erudite exposition. However, their stance risks complacency in that the notion of a crisis in psychiatric diagnosis was denied, and the authors sought to justify a conservative, incrementalist approach within the current paradigm. The corollary of their claim that no crisis exists is that no paradigm shift is required. In arguing their case, they highlighted the limitations of some of the candidates for a paradigm shift, notably HiTOP, Research Domain Criteria (RDoC; Figure 1), and network theory. However, they failed to consider the clinical staging model and other related elements of the psychiatric ecosystem. Incrementalism is always needed within paradigms but will fail to deliver progress if the paradigm is flawed, unproductive, and impedes progress. The fact that we have not succeeded yet in formulating a new improved paradigm is not a valid defense of the current one.

While it is essential that the field evolves from the current flawed diagnostic system, it remains unknown what a new system will look like. So far no single emergent approach to psychiatric diagnosis yet satisfies all the different demands placed upon it (e.g. neurobiological, treatment, sociological, consumer-friendly). Kendler (Kendler, 2024) and Stein and colleagues (Stein et al., 2024) have considered how we might attempt to integrate multiple perspectives. The optimal way to proceed is unclear. One alternative is to identify and evolve multiple heuristic systems or strategies that address different purposes and work to integrate these. Such an ecosystem of compatible and complementary approaches, which collectively enhance utility across different domains, could create the conditions for a true paradigm shift, or at least a stepping stone beyond complacency. This is an example of ‘integrative pluralism’ (Kendler, 2024). Another option would be to pursue ‘adversarial collaboration’ (Bateman et al., 2005; Rakow, 2022), which is an approach to resolving scientific disputes and paradigm clashes, wherein researchers who have different positions on the issue at hand collaborate with the aim of making progress on their disputed research question. This might result in the desired goal of integration or at least integrative pluralism, or it might lead to one of the heuristic models achieving preeminence based on new scientific data and superior utility and validity.

## Candidate pathways to a new paradigm for diagnosis

### Research domain criteria

RDoC is a translational research framework and is not a classification or diagnostic system. RDoC explores psychopathology as dysregulation in constructs jointly defined by data for a psychological/behavioral function (e.g. cognitive control or reward reactivity) and for an implementing neural circuit/system, rather than as symptom constellations defined *a priori* by clinical consensus (Figure 1) (Cuthbert, 2020, 2022). Constructs are viewed as dimensions that span the full range of population functioning from normal to so-called abnormal and cut across traditional disorder categories. Constructs are nested within broader domains of function, such as cognitive systems or social processes.

RDoC constructs are regarded as exemplars of the strategic approach, with novel or revised domains/constructs appearing continually as new data dictate. Research designs may involve one or multiple constructs (e.g. threat responses and attention). Emphasis is placed upon integrative analyses of multiple measurement classes (e.g. neurobiology, behavior, self-reports), and also upon studies examining developmental trajectories and environmental influences. Computational approaches are of high priority for using model-based paradigms to examine constructs defined by brain–behavior relationships (Viviani et al., 2020); for addressing heterogeneity and comorbidity with data-driven approaches to identify new transdiagnostic clinical phenotypes that share common mechanisms (Bzdok & Meyer-Lindenberg, 2018); and for identifying new treatment targets (Sanislow et al., 2019).

While RDoC has fostered studies that move toward precision psychiatry (Williams, 2020), its domains and constructs do not directly guide current clinical practice given its role as a heuristic research framework rather than a clinical diagnostic manual (although key scientific bodies have begun to discuss precision-medicine indications; National Academies of Sciences, 2016). Also, the goal of understanding psychopathology in terms of brain–behavior constructs, while promising for the long run, presents conceptual, experimental, and practical challenges at present. Critics, while acknowledging its cross-diagnostic strengths, regard RDoC not as an entirely new paradigm but more a rearticulation of preexisting ideas in biological psychiatry with limited clinical utility at this stage (Stein et al., 2022).

### Hierarchical taxonomy of psychopathology

The HiTOP consortium was formed by quantitative nosologists to integrate evidence from studies on the organization of psychopathology and develop a system based on these data (https://renaissance.stonybrookmedicine.edu/HITOP). The initial model has been published and is updated as data become available (Kotov et al., 2022; Kotov et al., 2021; Kotov et al., 2017; Krueger et al., 2018). The HiTOP system aims to address three limitations of traditional nosologies. First, it defines psychopathology in terms of dimensions of psychological function that range from normal to abnormal, resolving the problem of arbitrary boundaries. Second, HiTOP identifies dimensions based on observed covariation among signs, symptoms, and maladaptive behaviors, thus reducing heterogeneity within constructs. Third, it combines primary dimensions into larger spectra, thus recognizing comorbidity and capturing this in a hierarchical organization.

The HiTOP system includes over 100 fine-grained dimensions (e.g. insomnia, suspiciousness), larger subfactors, six broad spectra, and the general factor that contains features common to all psychopathology (Figure 1) (Caspi & Moffitt, 2018; Conway et al., 2019; Lahey et al., 2017). This system was derived from a large body of structural research, and many elements of it have been validated against genetic and neurobiological mechanisms, illness course, and treatment response (Conway et al., 2019; Kotov et al., 2020; Krueger et al., 2021; Waszczuk et al., 2020; Watson et al., 2022). Compared to traditional classification approaches, HiTOP has demonstrated better reliability and predictive power, and is gaining in acceptability to clinicians (Balling et al., 2023; Kotov et al., 2022; Kotov et al., 2020; Markon et al., 2011).

Recent papers have comprehensively summarized its progress and set out the agenda for evolving and enhancing HiTOP (Conway et al., 2023; Kotov et al., 2022; Kotov et al., 2021). This includes interplay with neurobiological research, improving research, clinical utility, and implementation in clinical settings (Kotov et al., 2022; Ruggero et al., 2019), and introducing a stronger longitudinal and developmental research perspective to transcend the largely cross-sectional nature of HiTOP, which has relied primarily on cross-sectional data and often in adults. HiTOP can provide useful phenotypes for longitudinal research, but the system does not yet include phenotypic features that sensitively reflect illness stage or course, and this aspect may be addressed in future research.

### Developmental approaches and clinical staging

Developmental research has revealed substantial heterotypic continuity, namely that psychopathology often evolves from one form to another with a variable level of accumulating comorbidity over a person’s lifetime (Caspi et al., 2020; Plana-Ripoll et al., 2019). This shows that static, cross-sectional approaches and hierarchical exclusion rules alone will not do justice to the diversity and complexity of the clinical phenotype.

Clinical staging aims to capture this dynamic, complex natural history and link it to pragmatic models successfully developed in other medical fields. Staging acknowledges the dimensional basis of psychopathology, recognizing complexity but proposing subcategories, the boundaries of which are defined by treatment needs and/or underlying neurobiological changes. Stein et al. (2022) point out that categorical and dimensional approaches are interchangeable: since not only can a dimension be converted into a category, but the reverse is equally true (Kessler, 2002).

Clinical staging thus attempts to define, especially at a first diagnostic encounter, nodes for where an individual is located at a given point in time along a continuum of illness (Figure 1) (McGorry & Hickie, 2019; McGorry et al., 2006). Clinical staging adopts a quasi-dimensional approach to multiple dimensions of symptomatology, delineating stepwise stage changes imposed upon continuous symptomatology to guide treatment decision-making, prediction, and aetiological research. Clinical staging takes a transdiagnostic approach by delineating illness stages *across* symptom domains and true (non-polythetic) syndromes (psychosis, mood, anxiety, etc.), rather than traditional disorders, which typically capture late-stage phenotypes, such as schizophrenia, which are further weakened by the confounds of variably coherent polythetic definitions. The latter often lack construct validity. This allows for a pluripotential mindset early in the course of a disorder where fluid heterotypy and frequent comorbidity are frequently present, as well as an inherent expectation of the evolution of symptoms over time.

The utility of clinical staging is that it mandates early treatment of distress and functional impairment, guiding this according to risk–benefit principles (Shah et al., 2022). That means treatments used earlier should prove safer, and be more effective than later. Later stages justify more risk and adverse effects since the stakes are higher. Longitudinal studies support this notion as later stages are associated with illness progression and poor clinical and functional outcomes (Capon et al., 2022; Iorfino et al., 2019). Staging is designed to *complement* syndromal diagnosis. Whether syndromal diagnosis complements staging depends on the capacity to show specificity of biological (or psychosocial) interventions, e.g. lithium for bipolar disorder. The evolution of clinical staging to clinicopathological staging, via the inclusion of pathophysiological biomarkers as in oncology, is part of the blueprint for precision psychiatry. Staging is in fact a heuristic framework that allows changes or stability in biomarkers to be studied and linked to (and potentially redefine) stage as well as syndrome (McGorry et al., 2014). However, one of the weaknesses of this more fluid approach, early in the course of illness especially, is that no definitive label can be offered, which some patients and families seek. This can reduce stigma and prevent premature closure; however, it can also be confusing and create anxiety.

### Comparison and integration

The three models have some common characteristics (Table 1). All include dimensions and emphasize the links between syndromes and behavior with biological variation. Clinical staging and HiTOP include categorization alongside dimensions. The distinctions and differing emphases complement each other. For instance, the powerful and sophisticated quantitative psychopathology techniques of HiTOP capture and organize dimensions of signs and symptoms in cross-section hierarchically. Clinical staging is compatible with this but adds both clinical and the potential for, biological, utility by adding a transdiagnostic fluidity and longitudinal dimension. In addition, it offers a heuristic framework for the interpretation and study of neurobiological markers and the conduct of clinical trials. RDoC characterizes these constructs in other units of analysis, offering a full biobehavioral description, including developmental trajectories (Ip et al., 2019), and prioritizes clinical research that could advance clinical utility in the future. Altogether, psychopathology can be understood as an ultimately (uni)dimensional construct (with subdimensions) that ranges from normal to dysfunctional, manifesting in biology as well as dimensions of behavior, which are all subject to change over time (Figure 1). However, full specification of constructs in three dimensions requires novel research designs and statistical methods. Furthermore, dimensionality must be channeled into new categories if clinical utility is to be achieved. Whether integration within a pluralist ecosystem is the final destination or a stepping stone to a new paradigm that may be more strongly derived from one of these candidate approaches is yet to be determined.

**Table 1.** Comparison of alternative models

	RDoC	HiTOP	Clinical Staging
**Characteristics**			
Type	Research framework	Classification system	Diagnostic framework
Dimensions	Yes	Yes	Yes
Classes	No	Yes (none yet specified)	Yes
Units included	Biology & behaviour	Behavior	Biology & behaviour
Hierarchically organized	Yes	Yes	No
**Focus**			
Computational description	Direct focus	Direct focus	Indirect focus
Normal development	Direct focus	Indirect focus	Direct focus
Illness course	Indirect focus	Indirect focus	Direct focus
Mechanisms	Direct focus	Indirect focus	Direct focus
**Strengths**			
Solutions to comorbidity and heterogeneity	Low	High	Moderate
Clarification of mechanisms and therapeutic targets	High	Moderate	High
Utility for clinical trials	Low	Low	High
Clinical utility	Low	Improving	High

*Note:* Type is either a system (specific set of features) or a framework (set of principles with more limited specification of features). Dimensions and classes are types of entities included (i.e. continuous or categorical). Units of analysis may include multiple modalities for measuring biology (e.g. genes, molecules, cells, circuits, and physiology) and behavior (e.g. self-reports, behavioral observations, and paradigms). Hierarchical organization describes relationships between constructs, such as when general constructs encompass specific constructs. Focus section lists features that are explicitly included in the model (direct) or are not yet explicated (indirect). Computational description is the statistical modeling of the construct. Normal development captures changes that occur prior to illness onset. Illness course captures changes that occur after onset. Mechanisms indicate specific causal processes. Clinical application indicates the extent to which the model can be used clinically at present.

## Research methodology to deliver a new paradigm

### Transdiagnostic and dimensional study designs

Future research should move away from traditional single disorder study designs, and adopt a transdiagnostic and a hybrid dimensional/categorical approach to psychopathology, which captures the full range of dysfunction at any of the axes displayed in Figure 1. New categorical divisions superimposed upon a background dimensional state selected through utility and validity would be expected to emerge. These would reflect a change in underlying biology or treatment need and efficacy. Future research will need to maximize sample sizes, e.g. through collaborative efforts, in order to capture sufficient variation across the different dimensions in Figure 1. Boundaries for sample selection will still need to be set for feasibility reasons but there may be a trade-off or ‘sweet spot’ that could be guided by staging. This approach was foreshadowed for the National Institute of Mental Health (NIMH) with the advent of RDoC but does not appear to have materialized, with research funding continuing to be allocated within the traditional diagnostic silos.

### The essential value of a developmental and staging perspective

There is an emerging appreciation of a lifespan view of psychopathology. About 75% of mental illnesses have an onset before the age of 25 years and the clinical phenotype is an evolving one; people tend to experience diverse mental disorders over the life course and every disorder is associated with elevated risk for every other disorder (Caspi et al., 2020; Kessler et al., 2005; McGrath et al., 2023; Solmi et al., 2022). Nonetheless, evidence to date for both conventional and proposed alternative approaches to classification of psychopathology is not currently developmentally informed or sensitive to illness stage (Kotov et al., 2017; Krueger et al., 2018).

The syndromal structure and biological substrates of psychopathology, as well as the interrelationships between the different axes in Figure 1, may differ at different developmental stages of onset and at different stages of disorder evolution. Future research designs should respect and capture the evolution of psychopathology over time against the context of normative human development (Cicchetti & Toth, 2009).

Novel methodologies with dense sampling over short periods of time (e.g. Ecological Momentary Assessments [EMA], actigraphy, or digital phenotyping) to complement less frequent, traditional assessments over longer time intervals should be employed. These methods, particularly EMA, could be aligned and applied within the coherent constructs defined via HiTOP (Simms et al., 2021). This would enable the validation, or alternatively the revision and reengineering of the existing HiTOP constructs within a longitudinal perspective. The results obtained within the cross-sectional studies may well be challenged by such longitudinal and developmental research designs and require amendment, which might become more congruent and better integrated with clinical staging. While the stability and replicability of network structures have been debated (Borsboom et al., 2017; Forbes et al., 2017), network theory is well-placed to guide this reengineering. Dynamic systems perspectives suggest that the extent and duration of the disordered state may undermine the resilience of the healthy state (Scheffer et al., 2024a, b). A corollary is that ‘Dynamic Indicators Of Resilience’ based on the pattern of fluctuation in any of the ‘units of analysis’ would be useful to monitor the risk of future disorder and quantify real-time treatment effects (Schumacher et al., 2023).

### Statistical methods to model the complexity of mental illness

The complexity of mental illness is increasingly acknowledged, as is the need to model this complexity (Maj, 2016; Nelson et al., 2017). While extensive work has been carried out using traditional multivariate techniques, several novel theoretical approaches and accompanying statistical techniques have emerged or been adapted from other fields that can assist in this development (Panel 1; Table 2). These involve capturing the dynamic nature of psychopathology over time and discovering dimensions of psychopathology that cohere with other patient, phenotypic, and environmental characteristics (e.g. biology, treatment response, social adversity, and social support) that cross traditional diagnostic categories.

Panel 1. Finding thresholds along the psychopathology dimension

Dimensional models of psychopathology have to be somehow reconciled with the often binary process of clinical decision-making. Similar to cutoffs for dimensional measures of, for example, hypertension (systolic blood pressure of 140 mm Hg or higher) or obesity (body mass index of 30 kg/m^2^ or higher), thresholds can be imposed on dimensional classifications of mental illness for categorical decisions. A major advantage of applying thresholds to dimensional psychiatric classifications for categorical clinical decision-making, as opposed to defining psychopathology as categorical entities, is that thresholds can be flexibly adjusted along the dimension for different types of decisions and adapted when more data (evidence) become available or with new developments in available interventions or theoretical developments. Hence, the threshold to achieve benefit at the population level differs from that at the individual level. A high Number Needed to Treat (NNT) will reveal and expose that disconnect (Haslam, 2022). Moreover, in line with a clinical staging model, one can adopt a multi-threshold framework, with stepped models of care provided for different thresholds (e.g. based on severity, stage, biological alteration, or a combination of factors).

Thus, even though psychopathology is expressed dimensionally, this does not preclude the existence of meaningful thresholds. Our challenge lies in identifying these thresholds and predicting transitions thereof. Importantly, thresholds should be empirically defined based on external criteria such as side-effect profiles of available interventions or the likelihood of progression to more serious stages of the illness. We recommend that future research systematically vary thresholds within the same sample—and compare the different thresholds with respect to their predictive value and NNT for interventions for clinically relevant outcomes—to determine the level of symptoms (or a combination of symptoms and other units of analysis) that define a sensible threshold. Such research can provide a set of standardized definitions for thresholds and clinical decisions, which is crucial for the coordination of care among treatment providers and the development of treatment guidelines.

Statistical techniques such as normative modeling and dynamic systems modeling are promising to identify meaningful thresholds or cut points for clinical decision-making (Table 2). Normative modeling allows identifying deviations from a normative variation in association between, for example, specific symptoms and level of dysfunction. As many associations may be characterized by inverted U-shape relationships (i.e. either too little or too much is associated with a dysfunctional state) (Northoff & Tumati, 2019), it is important to include nonlinear associations in normative modeling studies. Natural points of discontinuity (i.e. transitions) between normal or subthreshold states and a clinically significant disturbed state can be identified with dynamic systems modeling (Table 2). In this approach, transitions are preceded by accumulating instability within the system (Scheffer, 2009; Van De Leemput et al., 2014). This instability can be monitored or inferred from early warning signals (e.g. increasing autocorrelation, variance, and cross-correlations) (Scheffer, 2009), suggesting that sudden transitions in mental health (‘tipping points’) are preceded by early warning signals. This process has been demonstrated in depression and bipolar disorder. For example, in a case study of depression, increased autocorrelation and variance of momentary measures of feeling down as well as associations between different mental states were observed as early warning signs before a clinically and statistically significant transition in depression (Wichers & Groot, 2016).

**Table 2.** Examples of statistical techniques

Statistical technique	Description	Dimensions of the alternative classification models (Figure 1) that can be captured with the method	Attitudes shaping statistical approaches
Factor analysis and principal component analysis	Data reduction techniques that allow capturing the variance in variables with a smaller set. Principal component analysis (PCA) creates one or more index variables from a larger set of measured variables using a linear combination (a weighted average) of the set of variables. Factor analysis aims to find latent variables based on a larger set of variables and describes variability among observed, correlated variables in terms of a lower number of unobserved variables called factors.	Multiple variables within a single unit of analysis (e.g. symptoms).	Simplification and uncovering latent structures within data to understand underlying patterns and relationships.
Supervised machine learning	Supervised learning involves mathematical algorithms that learn the mapping function from input variables (X; e.g. a set of clinical, demographic, and biological measures) to an output variable (Y; e.g. onset of depression). The learning of the optimal function is usually done in a training phase and the optimal model identified in the training phase is subsequently tested on new data (either via cross-validation or a completely independent dataset) to evaluate how accurately the identified model can predict the outcome of interest. Many different mathematical algorithms for identifying the optimal mapping function exist (e.g. support vector machine, gaussian process, random forest, deep learning) and can be applied to predict either dimensional (i.e. regression) or categorical outcomes (classification).	Many different units of analysis can be accommodated in a single model to predict another unit of analysis, level of dysfunction, stage of illness, or changes over time (course, treatment).	Focuses on prediction accuracy and practical applicability, leveraging large datasets and complex algorithms to create robust predictive models.
Unsupervised learning methods	Unsupervised learning is when only input data (X) and no corresponding output variable (Y) are available and is used to model the underlying structure or distribution in the data. Unsupervised learning methods are commonly used to identify subgroups or subtypes of mental illness within the data, whereas factor analysis (see above) aims to group similar variables (instead of individuals) into dimensions. Popular unsupervised learning methods include clustering (e.g. hierarchical cluster, *k*-means clustering), which uses distance measures to identify groups or clusters in the data, and Finite Mixture Models (e.g. latent class analysis) that derive clusters using a probabilistic model that describes the distribution of the data.	Multiple variables within single units of analysis (e.g. symptoms) or level of dysfunction.	Aims to discover hidden patterns and natural groupings within data, emphasizing the exploration of data structures without predefined labels.
Canonical correlation analysis and partial least squares	These methods define linearly weighted composites of features in two datasets or data modalities (e.g. neural and symptoms) and choose a set of weights for each feature to maximize the association strength between the two datasets. These methods thus identify dimensions of maximum covariation between two data modalities. Each dimension represents an optimal mapping of how a set of features from one data modality covaries with a set of features from another data modality. If the association strength is measured by the correlation coefficient, the method is called canonical correlation analysis; if covariance is used the method is called partial least squares (PLS).	Any pair of units of analysis (e.g. symptoms and physiology) or pair of one unit of analysis and level of dysfunction. In case of high-dimensional data (e.g. genetic or neuroimaging data) regularized versions of CCA and PLS should be used to prevent overfitting.	Understanding and quantifying relationships between two sets of variables, often used to integrate and interpret complex, multimodal datasets.
Network modeling	Based upon the idea that symptoms are causally related over time. Clusters of symptoms that are strongly connected may form communities. Network estimation methods exist for cross-sectional (e.g. partial correlation, directed acyclic graphs [DAG], Ising model) and time-series data (e.g. several methods involving vector autoregressive [VAR] modeling: GIMME, mlVAR, graphical VAR).	Models interaction (multivariate) between multiple data points within units of analysis (e.g. different symptoms) or across units of analysis (e.g. symptoms and biological markers), both cross-sectionally and over time.	Understanding the dynamic and interrelated nature of variables, emphasizing the importance of interactions and dependencies in the data.
Joint modeling	Jointly models the trajectories of longitudinal risk factors, emerging symptoms and the risk of an outcome event, commonly utilizing Cox regression and linear mixed-effects modeling. Resulting model is useful in dynamic prediction of the outcome event.	Multiple units of analysis over time.	Aims to capture and predict the evolution of risk factors and symptoms over time, integrating various longitudinal data to enhance predictive power and dynamic understanding.
Normative modeling	Normative modeling is a dimensional approach that aims to model normative variation in the association between two or more quantitative measures (e.g. age and brain structure or level of dysfunction). Normative modeling provides statistical inferences at the level of the individual with respect to an expected pattern. This is analogous to normative growth charts in pediatric medicine to map child height or weight as a function of age with respect to centiles of variation in a reference population. Applying this to psychopathology, these variables can be substituted for clinically relevant variables then applying automated statistical techniques to map centiles of variation across the cohort. This has been used to map normal variation between any pair of cognitive, clinical, demographic, and quantitative biomarkers and allows identification of individuals that deviate from the normative range.	Two or more units of analysis (e.g. age and cognitive performance) or between a unit of analysis and level of dysfunction.	Highlights individual differences and deviations from the norm, providing a personalized context to understand clinical and biological variations.
Dynamical systems modeling	Based upon mathematical framework that uses differential equations to understand and predict how complex systems evolve and change over time. Behavior of systems can be predicted based on generic principles (including critical slowing down, critical fluctuations). Modeling network theory of psychiatric disorders using complex dynamical system methods has recently been performed in the context of panic disorder.	Multiple units of analysis over time.	Focuses on the temporal evolution and complex dynamics of systems, emphasizing prediction and understanding of how systems change and interact over time

A promising approach to modeling the structure of psychopathology is network analysis or modeling (Table 2). This is linked to dynamic systems theory and network theory (Table 2), building on the idea that mental disorders can be viewed as systems of causally interacting symptoms and other variables over time (Borsboom, 2017). The notion of mental disorders as a complex dynamic system aligns with many other scientific disciplines, such as ecology or meteorology, which have developed mathematical models for forecasting system transitions to different states in nature and even within financial markets. These tools have been applied to forecast transitions in mental disorders, such as depression and bipolar disorder (Bayani et al., 2017; Van De Leemput et al., 2014; Wichers & Groot, 2016). Preliminary evidence emerging from this work indicates that system-level early warning signals, such as critical slowing down, increasing autocorrelation, variance, and cross-correlations between symptoms (indicating increased system *instability*), might forecast transitions from healthy to disordered states (Helmich et al., 2021; Van De Leemput et al., 2014; Wichers & Groot, 2016). However, larger studies have also raised doubts about these findings (Bos et al., 2022; Curtiss et al., 2023; Helmich et al., 2023; Schreuder et al., 2023). This complex dynamic systems approach is consistent with network theory’s proposal that mental disorders can be understood as complex networks of mental states that trigger each other. In the scenario of high network connectivity, activation of a single node (mental state) may initiate a cascade of effects that resonate throughout the network. However, most research in network theory to date has been cross-sectional, and cross-sectional networks do not lend themselves to causal inference or prediction (Ryan et al., 2022). Therefore, network models have increasingly been applied to intensive longitudinal data, where temporal associations can be investigated (Borsboom et al., 2021; Bringmann, 2021; Bringmann et al., 2022).

The network approach can be easily reconciled with dimensional and transdiagnostic perspectives on mental illness. It provides more insight into the overall quasi-categorical structure of symptom interactions (e.g. which symptoms cohere and cluster stably together), as well as the role of individual symptoms (e.g. which symptom is most influential) (Borsboom, 2017; Borsboom & Cramer, 2013; Cramer et al., 2010). To define and map new syndromal patterns, data-driven, bottom-up approaches are essential, notably the study of dynamic processes within individuals at the idiographic level, and investigating to what degree these processes can yield more stable clusters across individuals (Beltz et al., 2016; Wright & Woods, 2020). The bridge between idiographic and nomothetic approaches with broader utility and links to other aspects of the new diagnostic ecosystem has yet to be crossed; however, network theory and analysis offer a possible ‘common vocabulary’ across disciplines and levels of analysis and an avenue to prediction and personalized therapies (Borsboom et al., 2021; Bringmann et al., 2022).

Another relatively novel statistical technique for modeling the dynamic nature of psychopathology is joint modeling. This technique jointly models the trajectories of longitudinal risk factors, including emerging symptoms, and the risk of an outcome event. Moreover, it enables dynamic prediction of the outcome (i.e. ongoing update of the risk estimate over time as further information about changes in symptoms and risk factors is obtained), which could guide personalized treatment (Illipse et al., 2023).

Supervised machine learning methods (Table 2) allow integration of multimodal data (Figure 1 and empirically identify the most relevant (combinations of) measures that predict outcomes over time. Future studies can help determine which units of analysis are most predictive of different course trajectories, clinical stages, or treatment outcomes. Large sample sizes and independent replication samples are critical for these types of studies to avoid overfitting (Vabalas et al., 2019; Varoquaux, 2018) and to ensure validity and generalizability of findings.

Finally, unsupervised machine learning algorithms are demonstrating their utility to incorporate a sizable number of variables in analyses with large cohorts, which can produce results that identify precision transdiagnostic phenotypes. The integration of multiple types of data adds increasing precision for deriving new psychopathological factors. Subgroups of patients identified by these analyses have already shown the capability of better prediction of treatment outcomes as compared to traditional disorders. With sufficient sample sizes for prediction, the promise of individualized treatment selection is already being tested (Bzdok & Meyer-Lindenberg, 2018). The US NIMH has begun to initiate such studies with a focus on clinical data, digital measures, and tasks to assess behavioral functions (Koutsouleris et al., 2021; NIMH, 2023).

## Stakeholders

There are many stakeholders in the diagnostic process: people with mental ill-health, their families, and funders of health care, notably governments, insurance companies, and other third-party payers. It is therefore critical to consider carefully the contexts in which diagnoses are currently used, how they are interpreted and what ‘work’ they do, their benefits and risks, and to engage with those most likely to be affected by change (see Panel 2 for a case vignette with a ‘sliding doors’ structure). This needs to include the notion of ‘dediagnosing’ to limit the effects of diagnoses that do not benefit people’s health or cause harm or waste of precious health care resources (Lea & Hofmann, 2022).

Panel 2. Vignette.

**Background**

Robin grew up with their younger sibling and single mother in supported housing in a large urban center in the United States. Robin is a member of a visible minority, speaks English and Spanish, and during childhood and adolescence the stability and security of their housing situation was fragile. Robin witnessed their mother being physically assaulted by their father from a young age and this domestic violence continued until Robin’s mother left the family home with the children when Robin was 10. Robin’s mother is a casual worker in the services industry. Her modest income covers the basics of the family’s expenses, but there is little financial security in terms of health insurance. Robin had suffered from anxiety during childhood but had functioned relatively well in primary school and had a number of close friends. However, problems began to surface in early high school. Robin became more anxious, and at times intensely distressed and overwhelmed. At age 14, Robin’s teacher had noticed that they had become ‘nervous’, quieter, and withdrawn. This began in the context of a prolonged period of being bullied. The teacher was aware of this and ensured that the school counselor provided support for Robin and that the bullying was eventually dealt with and ceased.

**Scenario 1: current diagnostic/system approach**

The school counselor provided a referral to a psychologist. At that first appointment with the psychologist, Robin was given a thorough evaluation and told that their symptoms were insufficiently severe and below the current threshold to meet criteria for any DSM disorder, which meant that they were not eligible to receive any age-appropriate services.

Robin was confused and somewhat frustrated by this, but returned to school for a few months and did their best to participate. However, when the feelings of anxiety, lowered mood, and irritability continued, Robin became slowly more and more discouraged, and their grades suffered. Robin eventually stopped attending school in their final year of high school, and withdrew socially from both friends and family, finding it increasingly difficult to interact with others. They spent more and more time in their bedroom playing video games at night and sleeping through the day.

A year later, Robin’s mother commented on this to a friend, an informal caregiver who was aware of community health clinics and provided a phone number to call. An appointment there was booked. Despite being reluctant to attend the initial visit, Robin went and was seen by a psychiatrist who noted that by then, Robin’s symptoms had changed and were characterized by prominent mixtures of anxiety and mood lability, yet still without reaching the threshold of a major DSM-5 diagnosis. Once again, because no specific diagnostic category seemed to fit and severity was below threshold, access to state-funded specialized care was not approved and the family lacked private medical insurance coverage.

Over the next couple of years, Robin began having distressing thoughts in the evenings that would cause difficulty in falling asleep, as well as nightmares of increasing frequency. Sometimes these were memories of the domestic violence they had witnessed as a child, although Robin did not feel able to discuss this with anyone. One of Robin’s friends suggested trying cannabis at night time, and Robin found this effective for sleep so began using this daily. Yet within weeks, first Robin and then their mother found them increasingly irritable and even irrational at times. They also found themselves becoming more suspicious and anxious in the company of others. They began to hear strange noises, and later whispering and mumbling, which sometimes became more distinct as actual verbal conversations. These ‘voices’ became frightening and critical, warning of danger and possible attacks. At first, these distressing experiences only occurred after smoking cannabis but later they became more intense and occurred at other times. Another new feature was instability of mood with fluctuations between deep depression and days of irritability, increased energy and confidence, and reduced sleep.

Robin’s distress and isolation increased and it became more difficult for their mother to communicate with them. They were less cooperative now with her attempts to get help for them. Now 19 years of age, they became more distressed and a sense of hopelessness and entrapment enveloped them and ultimately led to an overdose of their mother’s antidepressant medication. An ambulance was called and they were taken to the emergency department. They were admitted to an adult psychiatric unit where most of the other patients were in their mid-40s and had a diagnosis of schizophrenia. This was a very dispiriting and frightening experience for Robin. A diagnosis of drug-induced psychosis was assigned and antipsychotic medication was prescribed at doses which resulted in unpleasant side effects. After 5 days in hospital, the medication was abruptly ceased, based on the drug-induced diagnostic decision, and they were discharged with an appointment with the local general practitioner as the sole follow-up.

Over the next few months, Robin’s psychotic symptoms persisted and worsened, and further suicidal and at times aggressive behavior led to further hospital admissions and ultimately follow-up with the community mental health clinic. Robin was prescribed regular antipsychotic medication but received only minimal psychosocial support and their mother was largely excluded from appointments with the treating team, an approach explained on the basis of privacy and confidentiality. Robin by this stage was informed that their diagnosis had been changed to schizophrenia. They were confused and demoralized having had some exposure to what schizophrenia appeared to mean in the hospital and outpatient clinic for their future prospects.

**Scenario 2: new diagnostic approach**

The school counselor ensured that Robin was able to see a psychologist via the local integrated youth health service, which offered a warm, engaging welcome, a ‘listening ear’, and needs-based care for young people in the local community at an accessible stigma-free venue. No formal diagnosis was necessary to access care for their manifest distress and functional impairment, which involved peer support, psychological interventions to improve coping and reduce stress, and trauma-oriented care both for the bullying and childhood exposures. Robin was carefully followed up for several months as their mental health steadily improved and they continued at school with improved grades.

Later in high school, symptoms of anxiety and depression returned after a relationship breakup. Robin experienced a great deal of difficulty sleeping and began to be troubled by nightmares and memories of their violent childhood. They began using cannabis to manage these symptoms and help with sleep, but after a number of months, they began to develop panic attacks and feel frightened to go out because of increasing suspiciousness and fear of being harmed. They also began to hear strange sounds, which morphed into mumbling and whispering. Eventually, clear-cut voices and conversations distressed and disturbed them with hostile and critical themes, such as that Robin was in danger and was a terrible person who deserved to die. Mood instability was a new feature and days of deep depression were followed by days of increased energy and confidence, irritability, and reduced sleep.

Fortunately, Robin’s mother had noticed these changes, which had by now prevented them from attending school, and she and the school helped to arrange for the integrated youth health service to reach out to them once again. Because Robin was reluctant to venture out, the outreach worker (a clinical psychologist) came to the home to visit and carry out an assessment together with a youth peer worker. Because of their previous positive experience at the center, Robin was comfortable with this process and was able to re-engage with the team at the youth center. Due to the complex blend of short-lived and fluctuating symptoms in the context of cannabis use, the enhanced primary care clinicians at the youth center were reluctant to assign a definitive diagnosis but clearly recognized and communicated with Robin and his mother that a potentially serious condition had developed.

Robin was provided with a warm and personalized referral to the more specialized early intervention service where they were able to see a psychiatrist and gain access to a full multidisciplinary team oriented toward recovery. Investigations were carefully carried out to rule out other medical or central nervous system causes of the symptoms. The diagnostic term used to describe their presentation was first episode psychosis, as a ‘working diagnosis’, and low-dose antipsychotic medication was carefully prescribed along with anti-anxiety medication to ensure that Robin was able to sleep peacefully. Other interventions offered in sequence as they recovered were CBT and later exposure-based trauma therapy to refocus on earlier traumas. Vocational interventions helped them to return as soon as possible to education, and after recovery they were able to successfully complete high school and get a job. Robin’s drug use ceased as they felt less distressed and their other symptoms subsided, such that the drive to self-medication abated. Nevertheless, further acute relapses did occur later and the clinical picture became oriented more to one of mood disturbance with episodes of elevation of mood as well as periods of depression. Psychotic symptoms also returned but were less dominant. The treating team explained the descriptive and evolving nature of diagnosis to Robin and their mother early on, and they understood that treatments were tailored to syndromes and needs as they evolved, rather than a single traditional diagnostic label. Stigma appeared to be substantially minimized and a more hopeful stance to the future safeguarded through this approach .

**Summary**

The first scenario describes a pathway that is common under current diagnostic and service models. Timing of and access to care was dependent on clarity and severity of the diagnosis and quality was also affected. Robin was given a number of different conventional diagnoses to which they had various reactions, including feeling validated (anxiety and depression) but also stigmatized (psychosis). Without a formal DSM diagnosis, Robin could not really access evidence-based treatment due to the constraints of the funding of mental health care. The role of trauma and the value of psychosocial interventions were overlooked. The second scenario illustrates some of the advantages of a more flexible and agnostic approach, which recognizes the evolving nature of the clinical picture and the need for expert, holistic, and team-based mental health care during the earlier stages of illness before clarity and stability of the syndromal picture have been achieved or indeed so that such evolution can be halted in a proportion of cases.

Although the boundary between normality and mental ill-health is difficult to define, a decision to offer treatment or not must be made. Who decides? And on what basis? Is a diagnosis necessary or helpful? In addition to ‘objective’, yet arbitrary criteria, the person experiencing mental distress should have their say in when help can be expected and the diagnostic process. Clinicians and funders should acknowledge this. The assignment of a diagnosis is often equated with a ‘need for care’, though this is not necessarily the case (Lea & Hofmann, 2022). Because diagnoses may influence identity and human rights, the process should be explicitly discussed and negotiated with the patient (Lea & Hofmann, 2022). Need for care, as reflected in distress, risk, and/or functional impairment, typically precedes a traditional DSM or ICD diagnosis or ‘macrophenotype’. Clinicians should explain the syndromal basis and meaning of a diagnosis, and that early in the illness neither fixed or clear-cut diagnoses nor prognoses cannot necessarily be provided. Working or provisional diagnoses may be more useful and flexible, and suffice as guides to treatment options, including the decision that no diagnosis and no treatment are necessary.

Not being assigned a diagnosis may be confusing, frustrating, and stressful, but equally, a lack of diagnosis may be reassuring as long as it does not deny care. During the early weeks of treatment, a ‘working diagnosis’, complemented by a personalized formulation, which includes unique personal and contextual features, can become a focus around which to build a therapeutic alliance. If the clinical presentation is initially subtle or complex but then evolves, both clinician and patient should see that it would have been problematic to prematurely offer a hard or fixed diagnosis. This approach also reduces confusion caused by abrupt changes in diagnostic terms used, and may reduce the need for later ‘dediagnosis’ (Lea & Hofmann, 2022). The evolution of clinical presentation is consistent with the known natural history of mental illness and is not ‘failure’ by the original clinical team. What these changes mean is presently unclear. Do they have a single illness with an evolving clinical picture or are they attracting additional layers of complexity or comorbid syndromes? Such changes indicate a need to rethink and recalibrate the pattern of care required.

Even with the many limitations of current diagnosis, a large subgroup of people still find being able to put a name to their condition validating. Being able to name the condition in shorthand can demystify the whole experience and help people to communicate it to others. It can also generate vital access to practical, online, and social support and to welfare benefits. The field has also built a framework of hard-won clinical knowledge and evidence-based treatments around established diagnostic concepts, meaning that careful consideration will need to be given to how to salvage and retain what we already know from earlier clinical trials within traditional diagnostic boundaries within any new transcendent model.

## Challenges for a new paradigm

If after a wave of innovative research, a new paradigm were to emerge as a serious contender to supplant the current systems of psychiatric diagnosis, a number of daunting practical considerations would need to be addressed. A systematic worldwide educational process would have to be formulated and offered to existing clinical practitioners and introduced into the education of new graduates particularly in psychiatry and psychology but in fact across the whole of mental health care. There would be substantial impacts on health financing, on the legal system, which emphasizes diagnostic clarity above validity and flexibility, on welfare systems, and on systems for producing, regulating, and licensing new therapies. A comprehensive bridge and crosswalk would need to be developed between the former and the new system to ensure the vast body of existing research data was not rendered obsolete or irrelevant. This is a task that might require the power of Artificial Intelligence to address and master. The effort and expense of such a sea change could only be justified if the new paradigm conferred very substantial benefits in validity, utility, and acceptability to patients and the wider community. This raises the question of how such a judgment could be made, what kinds of research and data would be required, criteria to determine when data in support of such a new paradigm would be sufficiently persuasive that the effort and cost are justified.

## Conclusion

Diagnosis that works for the patient, the family, the clinician, and the researcher needs to be as simple as possible, but no simpler. The current approach to revisions of the existing diagnostic manuals, rooted as they are in the psychiatry of traditional tertiary care and opinion-driven consensus, will not reinvent diagnosis. Nor will endless introspection on the theme of diagnosis and its discontents, or a simple recycling of quantitative nosology from a static and purely psychopathological perspective. The field needs testable new models that are parsimonious enough to work in the clinic and yet complex enough to understand the underlying complexity of mental illness as well as support more personalized and sequential treatment selection. A perspective that links the tertiary care perspective to the modern and more inclusive population-based and primary care context may be best suited to take up the challenge of modernizing the diagnosis of mental disorders from first principles. This longitudinal approach can certainly be enhanced by quantitative nosology, clinical staging, network analysis, and dynamic biomarkers as potent research and design tools. The most important benefit to be gained is greater utility and a clearer and wider pathway to the holy grail of validity. This should moderate, redefine, and condense the ever-increasing generation (and occasional extinction) of diagnostic categories, by allowing the timing, and mode and extent of progression of illness to anchor the diagnostic process, and forge a stronger link to treatment decisions sensitive to risk–benefit considerations and patient choice.

A crucial step in constructing such a novel diagnostic strategy is to operationally define the early clinical phenotypes, which require intervention in their own right, but also connote risk for later stages and more elaborated syndromes, which are likely to be multiply comorbid and more persistent, recurrent, and disabling. The early clinical phenotypes may be initially truly pluripotential, or there may be early hints or warning signs, emerging symptom relationships and biosignatures suggesting particular patterns, sequences, trajectories, and outcomes. Treatments may also be characterized by cross-diagnostic effectiveness and preventive or preemptive influence and, at the same time, have specificity for certain aspects as well. These considered conjectures require a decisively heuristic approach to the early course and treatment of mental disorders. The holy grail is a single integrated model that is sophisticated and yet clear enough to meet the needs of patients, clinicians, researchers, policymakers, and society as whole. We believe this is an achievable dream but one that will depend on a new wave of innovative data-driven research, the evolution of the concepts and strategies described, and the influence of the consumers and funders of mental health care.
